# A system biology approach to understanding the molecular mechanisms of Gubentongluo decoction acting on IgA Nephropathy

**DOI:** 10.1186/s12906-016-1268-9

**Published:** 2016-08-24

**Authors:** Peicheng Shen, Jiaojiao Shen, Chuan Sun, Xuejun Yang, Liqun He

**Affiliations:** 1 Department of Nephrology, Shuguang Hospital Affiliated to Shanghai University of Traditional Chinese Medicine, 528 Zhangheng Road, Pudong Shanghai, 201203 People’s Republic of China; 2 Shanghai Key Laboratory of Traditional Chinese Clinical Medicine (14DZ2273220), Shanghai, People’s Republic of China; 3 Shanghai University of Traditional Chinese Medicine, Shanghai, People’s Republic of China

**Keywords:** Traditional Chinese Medicine, Gubentongluo decoction, IgA Nephropathy, Proteins, Network

## Abstract

**Background:**

Traditional Chinese medicine (TCM) has been widely used in treating various diseases in eastern Asia for several thousand years, and is becoming increasingly popular in western countries. Gubentongluo (GBTL) decoction, as a classic TCM formula, is commonly applied to treat IgA Nephropathy (IgAN) in China. To date, however, the pharmacological/molecular mechanisms of GBTL have not been fully elucidated.

**Method:**

In the present study, we used a system biological approach to explore these mechanisms acting on IgAN.

**Results:**

First, we found 3876 potential target proteins for GBTL (based on TCMID) and 25 known IgAN associated biomarkers (based on the OMIM or IPA database).16 of the latter biomarkers were direct targets of 6 of the 9 herbs in GBTL, suggesting that these components play a vital role in treating IgAN. Second, we showed that these 6 herbs mainly regulate the immune system and renin-angiotensin system, imbalance in which is the main factor leading to IgAN. Importantly, HUANG QI links with 14 biomarkers, indicating that it is the most important herb in GBTL for treating IgAN. Also, relationships of other herbs with IgAN were explored. Third, we demonstrated that the remaining 9 IgAN associated proteins are responses to biological processes, such as antigen processing, protein ubiquitination and cell cycle regulation, which are crucial for IgAN development. Finally, we found that GBTL could induce a significant increase in the levels of two target gene: TNF and NOS2.

**Conclusions:**

Further studies are called to develop/modify the formula of GBTL, in order to enhance its effect on IgAN.

**Electronic supplementary material:**

The online version of this article (doi:10.1186/s12906-016-1268-9) contains supplementary material, which is available to authorized users.

## Background

Traditional Chinese medicine (TCM), as a system of ancient medical practice, has been widely used in treating various diseases in Eastern Asia for many years. TCM continues to play a critical role in maintaining health for the peoples of China, and is growing in popularity in Western countries [1]. Notably, modern medical researchers have adopted ideas from TCM, using combinations of drugs to treat complex diseases such as cancer and diabetes.

In last decades, researchers have made great efforts to investigate TCMs and to analyze their components. To date, numerous bioactive ingredients of TCMs have been isolated and identified [2]. This has furthermore led to the discovery of a variety of single compound-based therapeutics in TCM, such as the anti-cancer compound salvicine [3]. Indeed, these data/database can provide important hints/information for further biological systematic studies, however, TCM protocols should take a more holistic method; the TCMs always works as the result of synergistic interactions by multiple ingredients [4]. Thus, to truly investigate TCMs, biological systematic methods should be applied, all the ingredients of herbs or formulae in TCM should be taken into consideration simultaneously [e.g. 5–7].

Currently, the most applied strategy in drug discovery is ‘one gene–one drug–one disease’ paradigm, involving the screening of potential compounds for individual disease-causing targets. In this case, the drug’s efficacy is often impaired, because the robustness of the protein interactions in the treated objectives was ignored. Therefore, the biological systems-oriented approaches, such as combinations of effective drugs with multiple targets, were called in drug discovery [8]. The TCM presents a perfect example, as it treats diseases in a holistic way. In the formulae of TCM, over hundreds compounds were always be treated simultaneously to rebalance the organism. Therefore, TCM formulae with multi-components/targets should be analyzed by the same strategy as combination therapies of multi-component drugs.

IgA Nephropathy (IgAN), a primary glomerular disease, is the most common type of Chronic Kidney Disease (CKD) which is a common condition affecting up to 11 % of the population. This disease is now recognized to elevate the risk of cardiovascular disease as well as kidney failure and other complications [e.g. 9, 10]. It is a leading cause of end-stage kidney disease in China [11]. Effective control of proteinuria may be a key strategy for treating CKD [reviewed in 12]. Reninangiotensin-aldosterone system blockers, glucocorticoids, and immunosuppressants have often been used for primary glomerular diseases. Immunosuppressive therapies have usually been applied to treat patients with heavy proteinuria, but are not entirely suitable for patients with non–nephrotic-range proteinuria [13]. Moreover, treatments with glucocorticoids and immune-suppressants are usually long term, which can result in severe adverse effects and increase the risk of rebound [e.g. 13, 14]. TCM has promising effects on the control of proteinuria, protection of kidney function, and improvement in patients’ clinical symptoms [e.g. 15, 16]. For example, *Abelmoschus manihot*, a traditional Chinese herb, has increasingly been used to treat a wide range of types of CKD, such as immunoglobulin A (IgA), nephropathy (IgAN) and diabetic nephropathy [e.g. 17, 18]. Clinical studies have proven that *A. manihot* can reduce proteinuria and thus protect kidney function [e.g. 19]. However, TCM formulae are multi-component and multi-target agents, and it is therefore necessary to investigate the combination therapy of multi-component drugs.

Gubentongluo (GBTL) decoction, a classic TCM formula from Chinese medical sage Zhang Zhongjing, is prepared from a basic formula of nine herbs, including *Rhizoma imperatae* (BAI MAO GEN), *Ramulus euonymi* (GUI JIAN YU), *Yerbadetajo herb* (HAN LIAN CAO), *Fructus ligustri lucidi* (NU ZHEN ZI), *Astragali radix* (HUANG QI), *Semen persicae* (TAO REN), *Rumex madaio* (YANG TI GEN), *Herba lycopi* (ZE LAN YE) and *Radix salvia miltiorrhizae* (DAN SENG). It is widely used in China in accordance with the China Pharmacopoeia standard of quality control. In TCM theory, the multiple agents contained in a single formula must work synergistically. With regard to GBTL, *Rhizoma imperatae* is the primary herbs and is believed to be a very effective antioxidant, whereas *Ramulus euonymi* acts primarily as an anti-inflammatory. For the most part, however, it’s the pharmacological/molecular mechanisms of GBTL have not yet been fully elucidated.

In this study, we have developed a comprehensive systematic approach for understanding the pharmacological mechanisms of GBTL acting on IgAN; an overview of our approach is shown in Additional file 1: Figure S1. Three steps were taken to achieve this objective: (1) prediction of potential targets for GBTL; (2) collection of IgAN associated molecules and construction of an IgAN associated regulation network; (3) study of the relationships of GBTL potential targets with the network and corresponding signal pathways and (4) examine whether the treatment of GBTL induces changes in potential targets expression. This procedure would enhance our understanding of the pharmacological mechanisms of GBTL and its limitations.

## Methods

### Description of herbs in GBTL and prediction of potential targets for GBTL and selection of IgAN-associated genes and proteins

The description of herbs in GBTL was obtained from the Traditional Chinese Medicine Integrated Database [TCMID, 5], which is the most commonly used noncommercial TCM database worldwide. In total, we collected information on nine herbs, i.e., *Rhizoma imperatae*, *Ramulus euonymi*, *Yerbadetajo herb*, *Fructus ligustri lucidi*, *Astragali radix*, *Semen persicae*, *Rumex madaio*, *Herba lycopi* and *Radix salvia miltiorrhizae*. Putative targets of the active ingredients (compounds) of each herb were identified potential in the TCMID, a comprehensive collection of herb ingredients’ targets based on articles published in both English and Chinese. The IgAN- associated genes and proteins were collected from two IgAN related databases, including OMIM (http://www.ncbi.nlm.nih.gov/omim [20] and IPA [21] (www.ingenuity.com).

### Protein-protein interaction (PPI) data

Protein-protein interaction (PPI) data were extracted from the STRING database version 9.1 (http://string-db.org/; [22]. Confidence scores for each pair of interacting proteins were calculated by combining probabilities from the different evidence channels, and correcting for the probability of observing random interactions. As these proteins don’t interact with each other directly, an integrated auto expanding algorithm was used to seek potential linking proteins between them. Among these expanded links, proteins that occur along the shortest path between the indirectly interacting pairs were kept to construct a fully connected network model.

### Pharmaceutical network construction and analysis

The PPIs between IgAN associated proteins were used to construct an IgAN associated regulation network. First, using Venn diagrams (http://www.omicsbean.com:88; Venn 1880), we identified: 1) overlapping proteins between the potential targets of each ingredient in GBTL and IgAN associated proteins (Group 1), 2) potential targets of each herb in GBTL which were not covered by the IgAN associated protein set (Group 2) and 3) IgAN associated proteins not included among the potential targets of each ingredient in GBTL (Group 3). Then, we explored the potential molecular mechanisms of GBTL acting on IgAN, by investigating the overlapping proteins between the potential targets of each herb in GBTL and IgAN- associated proteins. Finally, we tried to reveal the limitations of treating IgAN with GBTL alone, by studying the candidate proteins (of GBTL) which were not covered by the IgAN-associated protein set.

Furthermore, the herbs of GBTL, their potential targets, and the IgAN associated proteins were respectively used to construct an herb-potential target network, potential target PPI, IgAN associated protein PPI and herb-potential target-candidate IgAN target network. Cytoscape Version 3.1 [23] was applied to visualize the networks.

### *Gene Ontology (GO) and pathway enrichment analysis for* IgAN associated *proteins and potential targets of GBTL*

We used Database for Annotation, Visualization and Integrated Discovery (DAVID, version 6.7; [24] for GO enrichment analysis. The enrichment score was calculated using hypergeometric enrichment algorithms [25]. The EASE (Expression Analysis Systemic Explorer) score was set to the default value [26]. We also performed pathway enrichment analysis using pathway data obtained from the FTP service of KEGG (Kyoto Encyclopedia of Genes and Genomes, http://www.genome.jp/kegg/; [27]. *P*-values of the KEGG pathway were calculated using the Fisher exact test. Pathways with *P*-value <0.05 were taken as significantly enriched.

### Animal experiments

To examine whether the treatment of GBTL induces changes in potential targets expression, 7 male Haemophilus parainfluenzae antigens (OMHP) induced IgAN C3H/HeN mice were obtained from Shanghai Key Laboratory of Traditional Chinese Clinical Medicine (shanghai, china), and then randomly divided into two groups: the model group (*n* = 3) and GBTL-treated group (*n* = 4). The mice in GBTL-treated group were administrated with GBTL by intragastric at a dose of 10 g/kg bodyweight once a day for 3 days.

### Quantitative RT-PCR

After 3 days, all mice were killed and the kidneys removed from mice immediately were placed in liquid nitrogen. The kidneys were grinded into powder with pestle by adding liquid nitrogen. Total RNA was isolated from the powder of kidney (50 mg) using Trizol (Aidlab, China) by following the manufacturer’s instructions. Synthesis of single-stranded cDNA from 5 ìg of RNA was performed according to the “TUREscript 1st strand cDNA Synthesis Kit” from Aidlab (China), then, the mRNA was reverse transcribed into cDNA. Quantitative RT-PCR was performed using the QuantiTect SYBR Green PCR Mix (Qiagen) in a Roche LightCycler 480 II. The following primer pairs were used in this study to detect specific mRNAs, blinded to the treatment status of each sample: GAPDH: forward primer: GAG TCA ACG GAT TTG GTC GT; reverse primer: TTG ATT TTG GAG GGA TCT CG; TNF: forward primer: CCTGTAGCCCACGTCGTAG; reverse primer: GGGAGTAGACAAGGTACAACCC; NOS2: forward primer: AACCCCTTGTGCTGTTCTCAG; reverse primer: TGTGGCCTTGTGGTGAAGAG. Reaction conditions were set to 3 min at 95 °C (first segment, one cycle), 15 s at 95 °C and 60 s at Tm of a specific primer pair (second segment, 40 cycles) followed by 90 s at 90 °C, 60 °C for 3 min and 10s at 94 °C(Melting segment, one cycle) using Funglyn FTC-3000 (Funglyn Biotech). Relative gene expression was defined as a ratio of target gene expression versus â-actin gene expressionQuantification and comparisons of gene expression levels were performed using the − ÄÄCt method, and statistical analyses of differences between control and model and treatment group.

## Results

### Shared protein targets involved mainly in immune system process and regulation of renin-angiotensin system

**Fig. 1:**
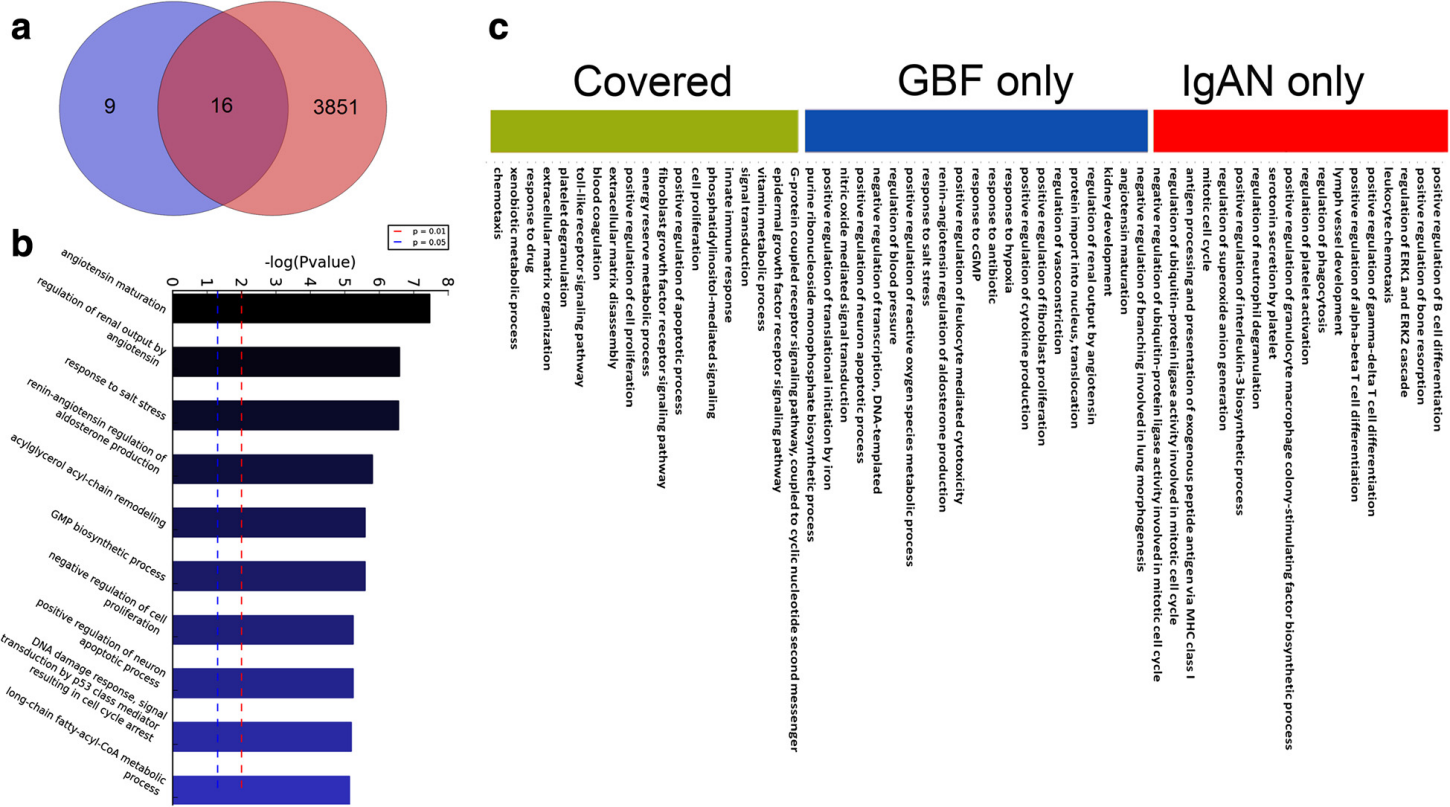
**a** Distribution of potential targets Gubentongluo decoction proteins and IgA Nephropathy - associated proteins, **b** Biological process enrichment result for 16 candidate proteins and **c** Heatmap of top 50 biological processes enriched in three groups. The color legend indicates association with the group, red means most relevant whereas the green indicates irrelevant

The 9 herbs of GBTL yielded 237 components and further resulted in 3867 potential proteins (list not shown), based on the TCMID database, whereas 25 IgAN associated proteins were detected from the OMIM or IPA database. Of the latter, 16 IgAN associated proteins, including ACE, TP53, JUN, TNF etc, were also determined to be the potential targets for GBTL (Fig. 1a; proteins listed in Table 1). It’s known that IgAN is associated with high levels of mononuclear leukocyte infiltration into the kidney [28], gene ontology enrichment of these proteins indicates that the leukocyte proliferation is the most relevant, with IMPDH1,IMPDH2, ACE, and TP53 involved. These genes were also proven to be associated with regulation of immune system process [29, 30]. Moreover, several important biological processes were found to be related to IgAN, such as angiotensin maturation, regulation of renal output by angiotensin and response to salt stress (Fig. 1b). These processes were linked to renin-angiotensin system which was the most used target systems for IgAN therapies [31], indicating that the pharmaceutical effects of the GBTL in treating IgAN might mainly through regulation of the leukocyte level and of the renin-angiotensin system, which could then regulate blood pressure and water balance in the IgAN patients.

**Table 1:** The list of 25 IgAN associated proteins

Uniprotein ID	Symbol	Name	Presence in GBTL
O43707	ACTN4	actinin, alpha 4	Yes
O75907	DGAT1	diacylglycerol O-acyltransferase homolog 1	Yes
P00797	REN	Renin	Yes
P01019	AGT	angiotensinogen (serpin peptidase inhibitor, clade A, member 8)	Yes
P01375	TNF	tumor necrosis factor (TNF superfamily, member 2)	Yes
P04637	TP53	tumor protein p53	Yes
P05412	JUN	jun oncogene	Yes
P11473	VDR	vitamin D (1,25- dihydroxyvitamin D3) receptor	Yes
P12268	IMPDH2	IMP (inosine monophosphate) dehydrogenase 2	Yes
P12821	ACE	angiotensin I converting enzyme (peptidyl-dipeptidase A) 1	Yes
P20839	IMPDH1	IMP (inosine monophosphate) dehydrogenase 1	Yes
P35228	NOS2	nitric oxide synthase 2, inducible	Yes
P49721	PSMB2	proteasome (prosome, macropain) subunit, beta type, 2	Yes
Q00987	MDM2	Mdm2 p53 binding protein homolog (mouse)	Yes
Q96D42	HAVCR1	hepatitis A virus cellular receptor 1	Yes
Q96PD7	DGAT2	diacylglycerol O-acyltransferase homolog 2	Yes
O00151	PDLIM1	PDZ and LIM domain 1	No
P11836	MS4A1	membrane-spanning 4-domains, subfamily A, member 1	No
P20618	PSMB1	proteasome (prosome, macropain) subunit, beta type, 1	No
P28074	PSMB5	proteasome (prosome, macropain) subunit, beta type, 5	No
P35612	ADD2	adducin 2 (beta)	No
P43405	SYK	spleen tyrosine kinase	No
Q13200	PSMD2	proteasome (prosome, macropain) 26S subunit, non-ATPase, 2	No
Q14005	IL16	interleukin 16 (lymphocyte chemoattractant factor)	No
Q99460	PSMD1	proteasome (prosome, macropain) 26S subunit, non-ATPase, 1	No

GBTL indicated the Traditional Chinese Medicine Gubentongluo decoction

### Functional analysis reveals GBTL could regulate innate immune response and inflammatory responses

Of 3867 potential targets of GBTL, 16 is overlapped with IgAN associated proteins, other 3851 proteins that are not directly linked to the IgAN (Fig. 1a). Figure 1c listed top 50 related biological processes of three groups of proteins (see methods), respectively. Processes, such as regulation of kidney development, cytokine production, renal output by angiotensin and hypoxia, are related to proteins in Group 1, as the core regulation mechanism to explain the effectiveness of GTBL. However, regulation of ERK1/2 cascade, chemotaxis, transmembrane transport, TLR signaling, innate immune response and inflammatory response are related to targets in Group 2. Indeed, Dys-regulated innate immune response and inflammatory response are likely causing failure of mucosal antigen elimination and IgA synthesis, and TLRs are relevant mediators of mucosal immunity [32]. Overall, GBTL could be able to regulate mucosal immunity and inflammatory responses.

In Group 3, we found that the 9 non-overlapping IgAN associated proteins play important roles in cell cycle regulation, protein ubiquitination and presentation of exogenous peptide antigen via MHC class I (Table 2). These pathways are all linked with the proteasome machinery [33].

**Table 2:** Biological processes of nine IgA Nephropathy associated proteins, which were not covered by Gubentongluo decoction

GO ID	Term	*P* value	*P* value FDR	Gene
GO:0006977	DNA damage response, signal transduction by p53 class mediator resulting in cell cycle arrest	4.29E-08	5.31E-04	PSMD1
				PSMB1
				PSMD2
				PSMB5
GO:0072413	signal transduction involved in mitotic cell cycle checkpoint	4.56E-08	5.64E-04	PSMD1
				PSMB1
				PSMD2
				PSMB5
GO:0051436	negative regulation of ubiquitin-protein ligase activity involved in mitotic cell cycle	4.56E-08	5.64E-04	PSMD1
				PSMB1
				PSMD2
				PSMB5
GO:0031571	mitotic G1 DNA damage checkpoint	6.83E-08	8.46E-04	PSMD1
				PSMB1
				PSMD2
				PSMB5
GO:0044783	G1 DNA damage checkpoint	7.21E-08	8.93E-04	PSMD1
				PSMB1
				PSMD2
				PSMB5
GO:0071158	positive regulation of cell cycle arrest	1.04E-07	1.28E-03	PSMD1
				PSMB1
				PSMD2
				PSMB5
GO:0016032	viral process	3.71E-07	4.59E-03	SYK
				IL16
GO:0002376	immune system process	7.59E-06	9.39E-02	ADD2
				SYK
				MS4A1
GO:0043306	positive regulation of mast cell degranulation	1.45E-05	1.79E-01	SYK
				MS4A1
GO:0051171	regulation of nitrogen compound metabolic process	4.45E-03	1.00E + 00	PDLIM1

### Core regulation network suggests HUANG QI is the key herb in GBTL

**Fig. 2:**
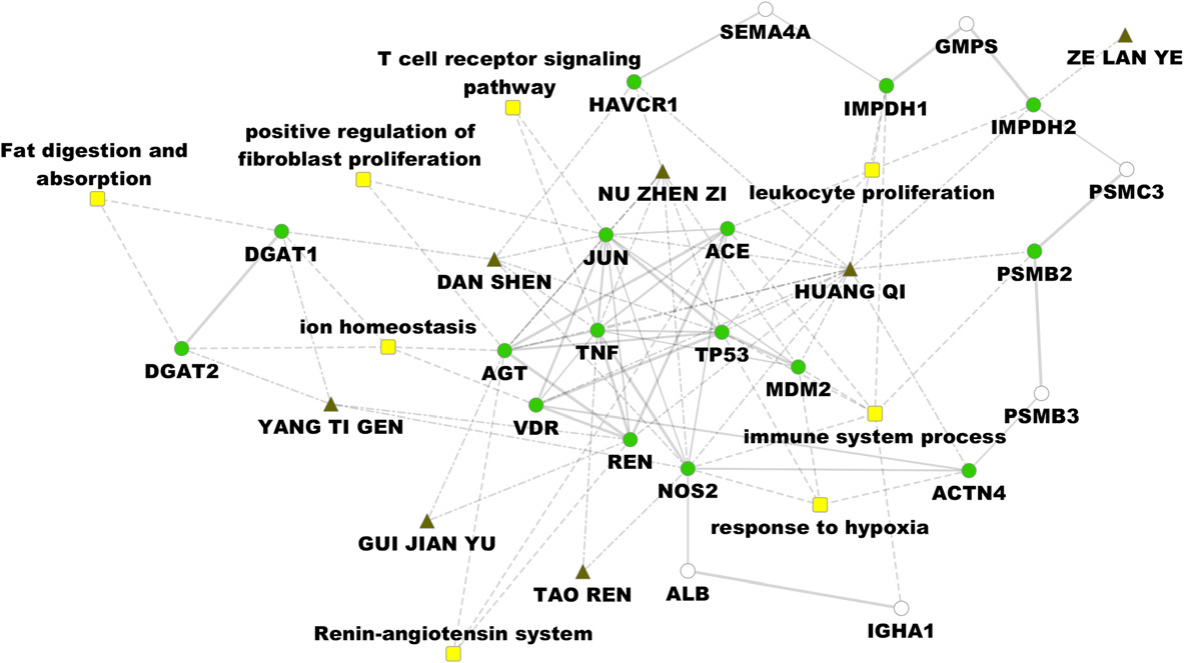
Core interaction network of components of Gubentongluo decoction on IgA Nephropathy. Triangle indicates the herbs, circle represent proteins, green colored indicates potential target, white colored indicates extended linking proteins, and yellow colored rectangle indicates related biological process. Solid line between two circles represents protein-protein interaction, the width of the line indicates confident score of the interaction; whereas the dashed line linked proteins to related processes

Based on PPI analysis, we found that 6 (i.e. GUI JIAN YU, HUANG QI, NU ZHEN ZI, ZE LAN YE, TAO REN, YANG TI GEN, DAN SHEN) out of 9 ingredients of GBTL acted on 16 associated proteins of IgAN (Fig. 2). Then, focus on the 16 covered biomarkers of IgAN, we have constructed a core regulation network. In this network, these 16 proteins linked different processes, including leukocyte proliferation, T cell receptor signaling pathway, renin-anglotensin system, response to hypoxia, fat digestion and absorption and immune systems process. HUANG QI is the most important herb in GBTL, as it contributes as a hub, connecting 14 target proteins. Based on the network model, HUANG QI could regulate leukocyte proliferation via IMPDH, ACE and TP53; the ion homeostasis via AGT and VDR, and hypoxia via ACTN4, MDM2 and NOS2. Indeed, it was showed that IgAN was associated with coupled up-regulation of iNOS and P53 that might linked with apoptotic activity of renal cells [34], suggesting that the hypoxia and P53 signaling are potential target pathway of GBTL. Moreover, these processes, which HUANG QI could be involved in, are linked with immune system processes with IgAN [35]. Therefore, the GBTL could regulate the immune systems and then act on IgAN.

The KEGG pathway enrichment analysis showed that these 14 proteins (i.e. the HUANG QI targeted in) were annotated in 20 different KEGG pathways, of which the most important pathway is the renin-angiotensin system. Moreover, the cell cycle and the Wnt signaling pathway are also highly associated. These pathways have been actively studied for the treatment of IgAN in recent years [36].

### Contribution of other herbs in GBTL

NU ZHEN ZI acts as the second hub in the network (Fig. 2), it could target both AGT (Angiotensinogen, an essential component of the renin-angiotensin system) and TP53. AGT is a potent regulator of blood pressure, body fluid and electrolyte homeostasis [37–39], whereas TP53 is key player in apoptotic activity in the pathogenesis of progressive IgAN [34]. This herb could also target JUN, a transcriptional factor, actives expression of IL-2, IFN and TNF in the T cell receptor signaling pathway, which further link to iNOS via HIF-1 signaling pathway.

DAN SHEN could target 6 proteins, 5 (JUN, TP53, TNF, NOS2, HAVCR1) of them are shared with HUANG QI or NU ZHEN ZI, suggesting it could have similar pharmaceutical effects as HUANG QI or NU ZHEN ZI. Another target is DGAT1, Diacylglycerol O-acyltransferase, a key metabolic enzyme converts diacylglycerol and fatty acyl CoA in the fat digestion and absorption pathway. This target is shared with YANG TI GEN, which targets not only DGAT1, but also DGAT2 (a homolog protein of DGAT1). Therefore, both the DAN SHEN and YANG TI GEN are responsible for synthesis of triglycerides. Indeed, increased level of triglycerides is a known nephritic syndrom in IgAN [40], thus, the GBTL may have potential effect on regulating the triglycerides level of IgAN patients.

The other two herbs, GUI JIAN YU and TAO REN, target AGT, REN and TNF, NOS2. The protein-protein interactions analysis showed that these proteins are interacted. AGT and REN are key roles in renin-angiotensin system, and NOS2 is induced by hypoxia. It’s known that the renin-angiotensin system could lead to hypoxia via induced oxidative stress, which causes directly endothelial cell damage [41]. Also, Hypoxia activates macrophage to express NOS2 and produce NO to lead to cell apoptosis [42]. The interacting network of AGT, REN, TNF andNOS2 could be a reasonable explanation that use combination of GUI JIAN YU and TAO REN could act on the upstream RAS system and downstream renal hypoxia simultaneously to provide better therapeutic effect on IgAN.

### EBV pathway is one of potential affected pathways of GBTL

**Fig. 3:**
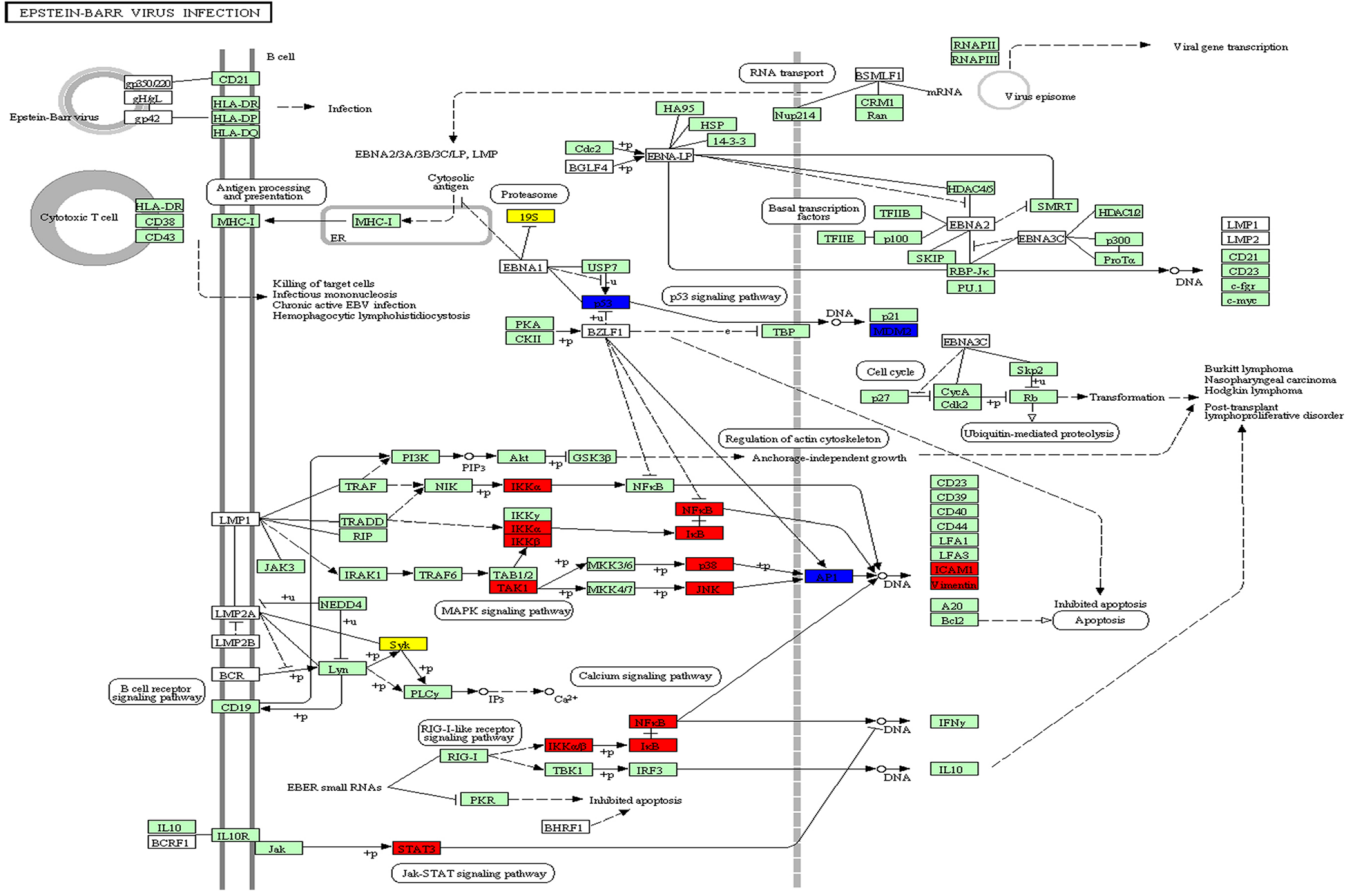
The pathway of Epstein-Barr virus infection affecting by the IgA Nephropathy associated proteins. The red and blue indicated the proteins covered by Gubentongluo decoction, whereas the yellow ones means uncovered in this formula of Traditional Chinese Medicine

Mesangial deposition of immunoglobulin A (IgA) is a main character of IgAN, and the up-regulated IgA level is positively associated with IgAN [43]. The Epstein-Barr virus (EBV) is a ubiquitous human herpes virus that is related with oncogenesis. A previous study has shown that EBV could up-regulate IgA in IgAN [44]. The EBV pathway was found to be the third most significantly associated pathway (*P* = 2e-0.5) with IgAN. Moreover, as shown in Fig. 3, the EBV pathway is regulated by 6 IgAN associated proteins (JUN, MDM2, PSMD1, PSMD2, SYK and TP53), 4 of them (JUN, MDM2, PSMB2 and TP53) are targeted by GBTL, suggesting that EBV pathway could be one of affected pathways in GBTL treatment. Two IgAN associated proteins (PSMD1 and SYK) were not covered by GBTL. SYK is known involved in downstream signaling of IgA1 stimulation, and serve as an important regulator to reduce the synthesis of MCP-1, IL-6,IL-8, and inhibit mesangial cell proliferation [45].

### GBTL treatment induced a significant increase in the levels of TNF and NOS2

**Fig. 4:**
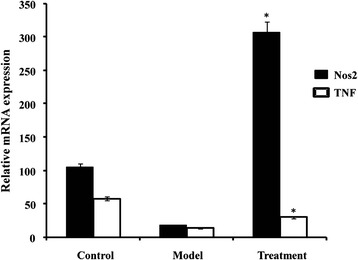
qRT-PCR analysis of relative expression level of TNF and NOS2 among three groups: Control: healthy mice, Model: OMHP induced IgAN model and Treatment: 3 weeks treated with GBTL. Data were normalized according to the β-actin expression level. * < 0.05

Two hub proteins (TNF: T cell receptor signaling, NOS2: response to hypoxia) for validation test were applied qRT-PCR with the kidney samples. GBTL treatment induced a significant increase in the levels of TNF (*p* = 0.046) and NOS2 (*p* = 0.018; Fig. 4).

## Discussion

In the present study, by using a systematic biology method, we investigated the molecular mechanisms of GBTL on treating the IgAN. Moreover, by using animal model, we found GBTL induced a significant increase in the levels of two target genes: TNF and NOS2. This provide an example for understanding the multiple targets of herbs in Traditional Chinese Medicine formulae and their interaction in the context of a molecular network [reviewed in 46]. Our work also indicated that a single TCM formula could be applied to the treatment of a given disease, since only parts of disease-based associated proteins were covered.

This work is the first study to show that the potential targets of GBTL could cover 16 of the 25 proteins which are believed to be associated with IgAN. This explains why GBTL is most effective for IgAN [e.g. [47–49]. Moreover, 6 of the 9 herbs of GBTL were determined to act on the 16 associated proteins of IgAN. HUANG QI is the most important component in the network, as it interacts with 14 IgAN associated proteins. Many previous studies have shown that HUANG QI can enhance myocardial contractility, improve circulation, protect myocardial cells and regulate immunity [e.g. 50, 51]. Our findings, in good agreement with a large number of other studies, indicate that HUANG QI has anti-viral, anti-inflammatory and immunoenhancing effects [e.g. 52–54]. Furthermore, by DGAT1and DGAT2, YANG DI GEN participates in the regulation of renin synthesis, which is associated with fat metabolism. In addition, NU ZHEN ZI, TAO REN and HUANG QI can be involved in T cell receptor signaling regulation though the TNF and JUN pathways. Since GBTL combines these herbs, it can provide an effective treatment to IgAN.

Out of 25 selected biomarkers of IgAN, 9 proteins were not overlapped with the potential targets of GBTL. Enrichment analysis of these 9 proteins suggests that biological processes such as G1/S transition of mitotic cell cycle, regulation of platelet activation, regulation of actin filament are also related to IgAN pathogenesis, however, GBTL may not able to regulate these processes, at least not directly as can be seen from the data. It suggests that this formula might have some shortcomings in the treatment of IgAN and could be improved in the future.

Moreover, many studies have already suggested that some of the herbs used in Chinese medicine can interact with drugs, and can have serious side effects [e.g. 55, 56]. In this study, more than 3800 proteins were not associated with IgAN directly. Although we showed that some of processes such as innate immune response and inflammatory response should associated with IgAN according to previous studies, processes such as synaptic transmission and response to drug could be related to potential side effects of GBTL. In contrast, the aforementioned 9 IgAN associated proteins are involved in several key biological processes, especially related to proteasome machinery. Most importantly, we have shown that in the pathway of the EBV, key genes/proteins (PSMB1 and SYK) related to the proteasome and downstream IgA1 production, were not targeted by GBTL directly.

Three herbs, BAI MAO GEN, ZE LAN YE and HAN LIAN CAO, were not associated directly with the IgAN, according to the current protein datasets. However, we analyzed these herbs separately and revealed that Bai Mao Gen is mainly associated with pathogenic *E. Coli* infection, gap junction and phagosome, Ze Lan Ye regulate mainly galactose metabolism, whereas, Han Lian Cao is related with NOD-like receptor signaling, TLR receptor signaling and TNF signaling (data not shown). These pathways were believed to be correlated with IgAN [e.g. 57–59], indicating that these herbs should have additive or complementary effect together with other herbs in GBTL.

## Conclusions

In this study we have shown the molecular mechanism of GBTL acting on IgAN. The GBTL potential protein-IgAN associated protein interactions showed that 6 herbs in GBTL acted on 16 IgAN associated proteins, mainly through the renin-angiotensin system, regulate the leukocyte proliferation and hypoxia, which are responsible for epithelial cell damage and leukocyte infiltration. DAN SHEN and YANG TI GEN have potential power to regulate the triglycerides level via DGAT1 and DGAT2. This demonstrates the basic therapeutic mechanisms of GBTL in treating IgAN. Our study also indicated that GBTL could not cover all IgAN associated biomarkers, such as Syk, a key mediator relevant to IgA1 stimulation. Overall, our study was the first to explore the molecular network of GBTL acting on IgAN, and further studies are called for to develop the formula of GBTL and to enhance its effectiveness on IgAN.

## Abbreviations

CKD, chronic kidney disease; GBTL, Gubentongluo; IgAN, IgA Nephropathy; TCM, Traditional Chinese medicine

## Additional file

Additional file 1: Figure S1. Overview of data generation, processing and analysis. (JPEG 118 kb)
