# Supplementary figures and images for: A system biology approach to understanding the molecular mechanisms of Gubentongluo decoction acting on IgA Nephropathy
Source: BMC Complement Altern Med. 2016 Aug 24;16(1):312. doi: 10.1186/s12906-016-1268-9 (PMC4997663; doi:10.1186/s12906-016-1268-9)

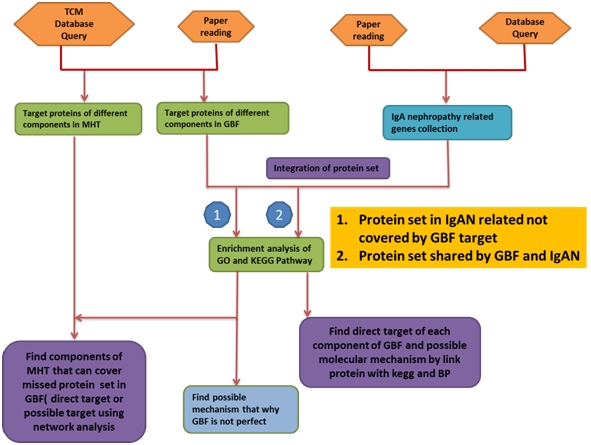

Supplement: Additional file 1: Figure S1. — Overview of data generation, processing and analysis. (JPEG 118 kb) [file 12906_2016_1268_MOESM1_ESM.jpeg]
